# Studies of Neotropical tree pathogens in *Moniliophthora*: a new species, *M.mayarum*, and new combinations for *Crinipellisticoi* and *C.brasiliensis*

**DOI:** 10.3897/mycokeys.66.48711

**Published:** 2020-03-30

**Authors:** Nicolás Niveiro, Natalia A. Ramírez, Andrea Michlig, D. Jean Lodge, M. Catherine Aime

**Affiliations:** 1 Instituto de Botánica del Nordeste (IBONE), Consejo Nacional de Investigaciones Científicas y Tecnológicas (CONICET). Sargento Cabral 2131, CC 209 Corrientes Capital, CP 3400, Argentina Consejo Nacional de Investigaciones Científicas y Tecnológicas Corrientes Argentina; 2 Departamento de Biología, Facultad de Ciencias Exactas y Naturales y Agrimensura, Universidad Nacional del Nordeste. Av. Libertad 5470, Corrientes Capital, CP 3400, Argentina Universidad Nacional del Nordeste Corrientes Argentina; 3 Department of Plant Pathology, University of Georgia, Athens, GA 30606, USA University of Georgia Athens United States of America; 4 Department of Botany & Plant Pathology, Purdue University, West Lafayette, IN, 47907-2054, USA Purdue University West Lafayette United States of America

**Keywords:** Agaricomycotina, fungal taxonomy, Marasmiineae, plant parasites, tropical fungi

## Abstract

The crinipelloid genera *Crinipellis* and *Moniliophthora* (Agaricales, Marasmiaceae) are characterized by basidiomes that produce long, dextrinoid, hair-like elements on the pileus surface. Historically, most species are believed to be saprotrophic or, rarely, parasitic on plant hosts. The primary morphological diagnostic characters that separate *Crinipellis* and *Moniliophthora* are pliant vs. stiff (*Crinipellis*) stipes and a tendency toward production of reddish pigments (ranging from violet to orange) in the basidiome in *Moniliophthora*. Additionally, most species of *Moniliophthora* appear to have a biotrophic habit, while those of *Crinipellis* are predominantly saprotrophic. Recently, several new neotropical collections prompted a morphological and phylogenetic analysis of this group. Herein, we propose a new species and two new combinations: *Moniliophthoramayarum***sp. nov.**, described from Belize, is characterized by its larger pileus and narrower basidiospores relative to other related species; *Moniliophthoraticoi***comb. nov.** (= *Crinipellisticoi*) is recollected and redescribed from biotrophic collections from northern Argentina; and *M.brasiliensis***comb. nov.** (= *Crinipellisbrasiliensis*), a parasite of *Heteropterysacutifolia*. The addition of these three parasitic species into *Moniliophthora* support a hypothesis of a primarily biotrophic/parasitic habit within this genus.

## Introduction

The crinipelloid genera *Crinipellis* Pat. and *Moniliophthora* H.C. Evans, Stalpers, Samson & Benny are characterized by basidiomes that produce thick-walled, dextrinoid, hair-like terminal cells on the pileus surface (Kerekes and Desjardin 2009). These belong to the Marasmiaceae in a lineage that includes *Marasmius* Fr. and *Chaetocalathus* Singer (Aime and Phillips-Mora 2005; Antonín 2013). *Crinipellis* and *Moniliophthora* appear to be most speciose in the Neotropics (Singer 1976; Kerekes and Desjardin 2009). Only a few authors have studied these genera in the Neotropics, primarily Singer (1942, 1976), who described 41 neotropical species of *Crinipellis*.

*Moniliophthora* was described by Evans et al. (1978) as an *incertae sedis*, monotypic genus of basidiomycetes, with *M.roreri* (Cif.) H.C. Evans, Stalpers, Samson and Benny, a parasitic fungus of *T.cacao*, as the type. Aime and Phillips-Mora (2005) used a five-locus analysis to place *M.roreri* within the Marasmiaceae (Agaricales), and included two additional species in *Moniliophthora*: *M.* (= *Crinipellis*) *perniciosa* (Stahel) Aime and Phillips-Mora – also a pathogen of cacao – and an unnamed species known only as an endophyte of the grass *Bouteloua* Lag. The authors speculated that other *Crinipellis* species, especially those currently placed in section Iopodinae (Singer) Singer, would be found to belong to *Moniliophthora* (Aime & Phillips-Mora, 2005). Subsequent studies have added an additional five species of mushroom-forming agarics to *Moniliophthora*: *M.aurantiaca* Kropp & Albee-Scott (Kropp and Albee-Scott 2012), *M.* (=*Crinipellis*) *canescens* (Har. Takah.) Kerekes & Desjardin (Kerekes and Desjardin 2009), *M.* (=*Crinipellis*) *conchata* (Har. Takah.) Antonín, Ryoo & Ka (Takahashi 2002), *M.marginata* Kerekes, Desjardin & Vikinesw., and *M.* (=*Crinipellis*) *nigrilineata* (Corner) Desjardin & Kerekes (Kerekes and Desjardin 2009). The primary morphological diagnostic characters that separate *Crinipellis* and *Moniliophthora* are pliant vs. stiff (*Crinipellis*) stipes, and a tendency toward production of pink to orange pigments in the basidiome, that do not change to green or olive when treated with KOH or NaOH (*Moniliophthora*). Additionally, many *Moniliophthora* species appear to have a biotrophic habit, including important pathogens of tropical crops such as cocoa (*Theobromacacao* L.), while those of *Crinipellis* are primarily saprotrophic.

Recent collecting efforts in northern Argentina and within the Mayan Mountains of Belize included two crinipelloid species. One, an orange fungus fruiting copiously from living roots and trunks of three different species of living trees in Argentina was identified as *Crinipellisticoi*. The other, an orange fungus fruiting gregariously on a dead root in Belize was determined to represent a new species of *Moniliophthora*. Herein we provide updated descriptions, as well as phylogenetic analyses supporting the placement of these and one other former species of *Crinipellis* within *Moniliophthora* as: *M.ticoi* comb. nov., *M.brasiliensis* comb. nov., and *M.mayarum* sp. nov., bringing the total number of known species of *Moniliophthora* to 11.

## Methods

### Morphological studies

The specimens studied here were collected in Belize (deposited at BRH and CFMR) and from northern Argentina (deposited at CTES). Specimens were described macroscopically according to Largent (1986). Kornerup and Wanscher (1978) colors are followed by chart numbers and letters in parentheses. Capitalized color names are from Ridgway (1912) as reproduced by Smithe (1975), except for Spectrum Orange which was created by Smithe (1975) to fill a gap. Microscopic characters were examined by light microscopy (LM) on a Leica model CME or an Olympus BH-2. All LM images were made with a Leica EC3 incorporated camera from material mounted in 5% KOH and Phloxine (1%), and Melzer’s reagent. The measurements were made directly in the LM or through the photographs taken using the software IMAGEJ (Schneider et al. 2012). Microstructures (length and width of spores, basidia, hyphae, pileipellis) were measured using LM. The following notations were used for spore measurement: *x* = arithmetic mean of the spore length and width, with standard deviation (+/-); Q = quotient of length and width indicated as a range of variation; Q*_x_* = mean of Q values; n = number of spores measured, N = number of analyzed basidiomes. All GPS readings were taken on a Garmin eTrex 10, hand-held unit using WGS84 standard. Herbarium abbreviations follow Index Herbariorum (Thiers 2019) and authors’ abbreviations follow Kirk and Ansell (1992).

### DNA extraction, amplification, and sequencing

Extraction, amplification and sequencing of the new species at CFMR in Madison, WI followed Lindner and Banik (2009). For the other specimens, DNA was extracted from dried basidiomes using the Promega Wizard Genomic DNA Purification Kit (Promega Corp., Madison, WI, USA). Amplification of the internal transcribed spacer (ITS) and large subunit (28S) of the ribosomal DNA repeat follow the methods of Aime and Phillips-Mora (2005). Sequencing of PCR products was conducted at GeneWiz (South Plainfield, NJ, USA). Sequences were manually edited with Sequencher 5.2.3 (Gene Codes Corp., MI, USA) and confirmed via BLAST queries of the NCBI databases (National Center for Biotechnology Information, Bethesda, MD, USA). Collection data and GenBank accession numbers of the specimens used in this study are detailed in Table 1.

**Table 1.** Origin of sequences used in this study.

**Taxon**	**Coll.** #	**Country**	**ITS**	**LSU**	**Source**
*Brunneocorticiumcorynecarpon*	MCA 5784	Guyana	MG717359	MG717347	Koch et al. (2018)
*Chaetocalathusliliputianus*	MCA 485	Puerto Rico	AY916682	AY916680	Aime and Phillips-Mora (2005)
*Chaetocalathus* sp.	MCA 2538	Ecuador	AY916686	AY916684	Aime and Phillips-Mora (2005)
*Crinipellis* sp.	MCA 2240	Guyana	MG717367	AY916695	Koch et al. (2018) (ITS); Aime and Phillips-Mora (2005) (LSU)
*Crinipellis* sp.	MCA 1527	Guyana	AY916701	AY916699	Aime and Phillips-Mora (2005)
*Marasmius* sp.	MCA 1708	Guyana	AY916720	AY916718	Aime and Phillips-Mora (2005)
*Marasmius* sp.	MCA 7492	Cameroon	MG717368	MG717354	Koch et al. (2018)
*Marasmiusrotula*	PBM2563	USA	DQ182506	DQ457686	Matheny et al. (2006a) (ITS); Matheny et al. (2006b) (LSU)
*Moniliophthoraaurantiaca*	UTC253824 ^T^	American Samoa	JN692482	JN692483	Kropp and Albee-Scott (2012)
*Moniliophthorabrasiliensis*	UB2053	Brazil	AY317137	–	Arruda et al. (2005)
*Moniliophthoracanescens*	DED 7518	Malaysia	FJ167668	–	Kerekes and Desjardin (2009)
*Moniliophthoramayarum*	DJL BZ511^T^	Belize	MT162718	MT162714	This paper
*Moniliophthoraperniciosa*	MCA 2520	Ecuador	AY916743	AY916742	Aime and Phillips-Mora (2005)
*Moniliophthoraroreri*	MCA 2953	Mexico	DQ222925	DQ222926	Phillips-Mora et al. (2006a)
*Moniliophthoraroreri*	MCA 2954	Belize	DQ222927	DQ222928	Phillips-Mora et al. (2006b)
*Moniliophthora* sp.	MCA 2500	USA	AY916754	AY916752	Aime and Phillips-Mora (2005)
*Moniliophthora* sp.	MCA 2501	USA	MT162719	MT162715	This paper
*Moniliophthoraticoi*	NY00511157^T^	Bolivia	MT162721	MT162717	This paper
*Moniliophthoraticoi*	Niveiro 2249	Argentina	MT162720	MT162716	This paper
*Tetrapyrgosnigripes*	MCA 6925	USA	MG717370	MG717355	Koch et al. (2018)

T = type material

### Phylogenetic analysis

Initially, sequences derived for this study were analyzed within a dataset (Aime unpubl.) of 612 published and unpublished Marasmiaceae sequences inclusive of all genera in the family (data not shown). Results from preliminary phylogenetic and blast analyses indicated that the Argentina and Belize material both belong within *Moniliophthora*, as does *Crinipellisbrasiliensis* – the sister species to *M.perniciosa* (Arruda et al. 2005). Datasets were then trimmed to include: 1) all species of *Moniliophthora* for which ITS and/or 28S sequence data exist (only ITS data were available for *M.canescens*, *M.aurantiaca*, and *M.brasiliensis*); 2) newly generated sequences of the material from Argentina and Belize and from the type of *C.ticoi*; 3) exemplar sequences from the other related Marasmiaceae genera – *Crinipellis*, *Marasmius*, and *Chaetocalathus* – for context (Aime and Phillips-Mora 2005; Antonín et al. 2014; Koch et al. 2018). Individual datasets for each locus were aligned in GENEIOUS 9.1.5 (Biomatters Ltd., Auckland, NZ) using the MUSCLE algorithm (Edgar 2004). Individual alignments were then concatenated in Geneious, and analyzed by maximum likelihood (RAXML; Stamatakis 2006) methods using the CIPRES Science Gateway (Miller et al. 2010), following the methods of Koch et al. (2018).

## Results

### Phylogenetic analyses

Based on our Maximum Likelihood (ML) analysis of ITS and 28S rDNA, Marasmiaceae is comprised of two clades, *Marasmius* + *Crinipellis* + *Moniliophthora* + *Chaetocalathus* and *Tetrapyrgos* + *Brunneocorticium*. *Moniliophthora* is the sister genus to *Crinipellis* (96 BS).

**Figure 1.**
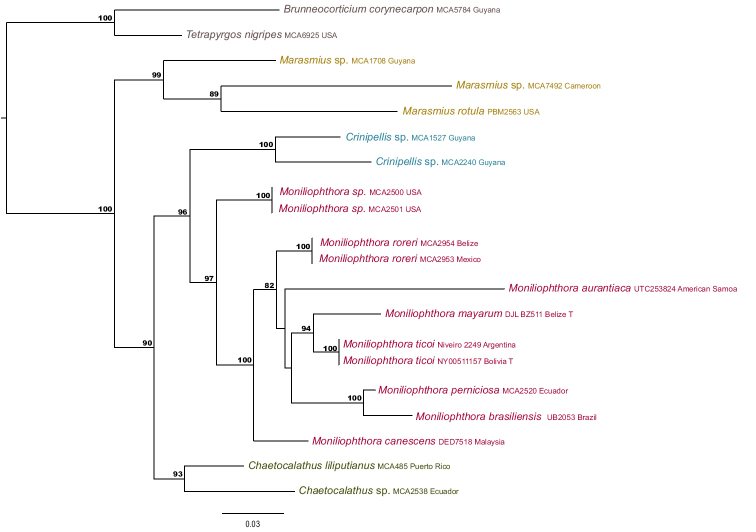
Maximum likelihood (ML) tree of Marasmiaceae based on dataset of ITS and LSU sequences. Bootstrap values above 50% are shown at supported node. **T** indicates type material. The tree was rooted with *B.corynecarpon* and *T.nigripes* (Aime and Phillips-Mora 2005; Koch et al. 2018).

The newly sequenced material (*M.mayarum* and *M.ticoi*) are strongly supported as members of *Moniliophthora*, as is *C.brasiliensis* based on previously sequenced material (Arruda et al. 2005) (Fig. 1). The collected material in Argentina shared 100% identity with the type specimen of *Crinipellisticoi*, collected and described from Bolivia.

### Taxonomy

**New species**

#### Moniliophthora mayarum

Taxon classificationFungiAgaricalesMarasmiaceae

Lodge, Aime & Niveiro sp. nov.

4B6AA2C3-D9E4-5B05-B9C1-DF92F084AECC

830319

Genbank No: MT162718 (ITS), MT162714 (LSU)

Figs 2A , 3A–F

##### Diagnosis.

*Moniliophthoramayarum* differs from *M.aurantiaca* and *Crinipellishygrocyboides* by larger pileus (> 15–20 mm) and narrower basidiospores (3.2–4.2 vs > 4–6 µm). Differs from *M.ticoi* by smaller basidiospores (8.0 +/-1.3 × 3.8 +/-0.3 µm vs 12.1 +/-0.8 × 5.4 +/-0.4 µm).

##### Type.

Belize, Stann Creek District, Cockscomb Basin Wildlife Sanctuary, Jaguar Preserve, near Maya Center Community, Rubber Tree Trail, on dead tree roots, possibly *Ceibapentandra*, 16°42'58.32"N, 88°39'38.88"W, 180 m a.s.l., 16. 11. 2001, D.J.Lodge, K.K.Nakasone, S.Schmeiding, E.Gaitlan BZ-43-Nov-2001, BZ-511 (**Holotype**: CFMR!)

##### Description.

***Pileus*** 7–20 mm, convex with an inrolled margin when young, broadly convex with age, some slightly depressed at center, some with a papillate umbo, color Chrome Orange (Plate II, 11, -), with center Scarlet (Plate I, 5, -) to Flame Scarlet (Plate II, 9, -), surface moist or slightly viscid when wet but not gelatinized, smooth, rarely sparsely minutely pubescent on umbo when dry, margin translucent-striate to disc, some sulcate-striate with age. ***Lamellae*** subdistant, 2 per mm on margin and half-way to margin, adnate or slightly adnexed, 2–4 mm broad, regular, 1 or more lengths of lamellulae inserted, Spectrum Orange with a coral tint, margin even, concolorous. ***Stipe*** central, 12–27 × 0.8–1.2 mm, equal or slightly clavate, some flared at apex, pale Spectrum Orange, pale Orange-Yellow (Plate III,17, f) at apex, surface dry, densely minutely pubescent, dense Warm Buff (Plate XV, 17´, d) mycelial pad at base. ***Annulus*** absent. ***Spore-print*** not observed, presumably white. ***Context*** pale orange in pileus and stipe, odor none, taste sweet. KOH and NaOH reactions on pileus surface negative.

**Figure 2.**
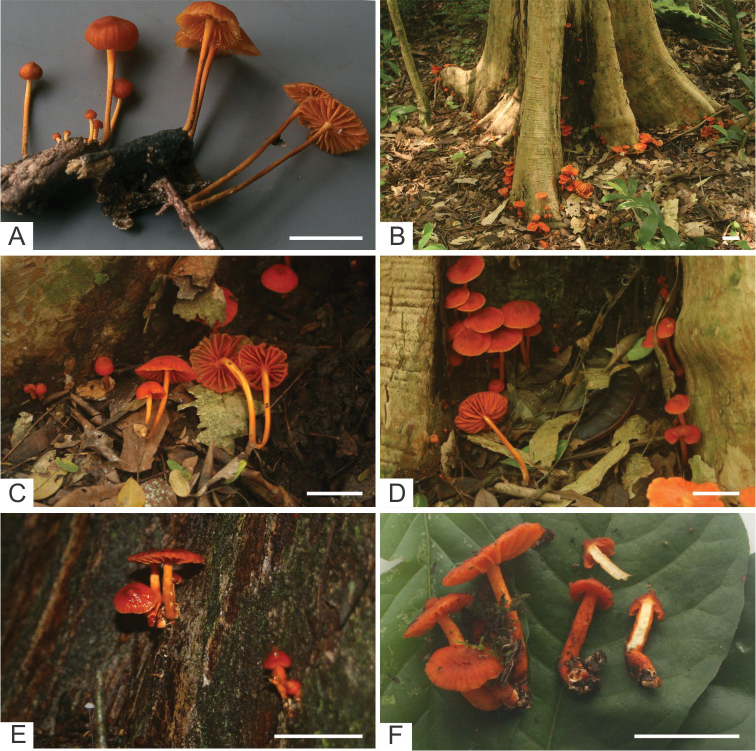
Photographs of sister species, *Moniliophthoramayarum* and *M.ticoi*: **A** basidiomes of *M.mayarum* on piece of tree root in Belize (BZ-511) (photo by S. Schmeiding) **B–F** Basidiomes of *M.ticoi* on trunks of *Holocalixbalansae* (Fabaceae) and *Pogonopustubulosus* (Rubiaceae) in Argentina. Scale bars: 10 mm.

***Basidiospores*** on lamellae of two sizes, larger ones 6.5–8.5(–10.5) × 3.2–4.2 µm, *x* = 8.0 +/-1.3 × 3.8 +/-0.3 µm, Q= 1.60–2.65, Q*_x_*= 2.02 +/-0.3, n=14; smaller spores 4–6 × 2.4–4.2 µm, *x*= 5.2 +/-0.8 × 3.3 +/-0.6 µ µm, Q= 1.25–1.89, Q*_x_*= 1.60 +/-0.3, n=10. ***Basidia*** 4-sterigmate, 14.4–28 × 4–8 µm, sterigmata up to 6.4 µm long, with basal clamp connections. ***Pleurocystidia*** absent. ***Cheilocystidia*** 22–26.5 × 6–13 µm, of three types: 1) clavate or hyphoid, 2) with 2–3 lobes, 3) clavate with apical digitate appendages or irregular lumps overall. ***Hymenophoral trama*** regular, hyphae 2.6–5.2 µm diameter, smooth, thin-walled, not dextrinoid, with clamp-connections. ***Pileipellis*** a cutis of repent, more or less interwoven hyphae, 4–8 µm broad, thin-walled ones occasionally with incrusted rusty pigments, apical segments of some hairs thick-walled and dextrinoid. ***Hairs of the pileus surface*** setiform, dextrinoid thick-walled part (66–)86–240 × (4.8–) 5.1–8.2 µm, comprised of 1–3 segments dextrinoid, walls (1.4–)2–4 µm thick, hyphae sometimes almost occluded, septa usually with clamp connections but clamp connections absent on the few secondary septations, with obtuse or acute apex. ***Hypodermium*** of short, broad, thin-walled cells 21.6–24 × 16–17.5 µm, with basal clamp connections.

##### Distribution.

Know only for the type locality.

##### Ecology.

Gregarious, putatively parasitic on roots of a tree, possibly *Ceibapentandra* (L.) Gaertn.

##### Etymology.

mayarum – of the Maya people in the region where the fungus was found.

##### Specimens studied.

Belize • Stann Creek District, Cockscomb Basin Wildlife Sanctuary, Jaguar Preserve, near Maya Center Community, Rubber Tree Trail, on dead tree roots, possibly *Ceibapentandra*; 16°42'58.32"N, 88°39'38.88"W, 180 m a.s.l.; 16.XI.2001; D.J.Lodge, K.K.Nakasone, S.Schmeiding, E.Gaitlan BZ-43-Nov-2001, BZ-511 (**Holotype**: CFMR!; Isotype BRH!).

##### Observations.

Few previously described *Crinipellis* and *Moniliophthora* species share the striking bright orange coloration of *M.mayarum*. This taxon most closely resembles *M.aurantiaca* Kropp & Albee-Scott described from the South Pacific island of Samoa, *Crinipellishygrocyboides* (Henn.) Singer (= *Marasmiushygrocyboides* Henn.) described by Hennings from Africa, and *M.ticoi* (Halling) Niveiro, Ramírez, Lodge & Aime described from South America. Our phylogenetic analysis places *M.mayarum* as a sister species to *M.ticoi*-the other Neotropical species in this complex.

**Figure 3.**
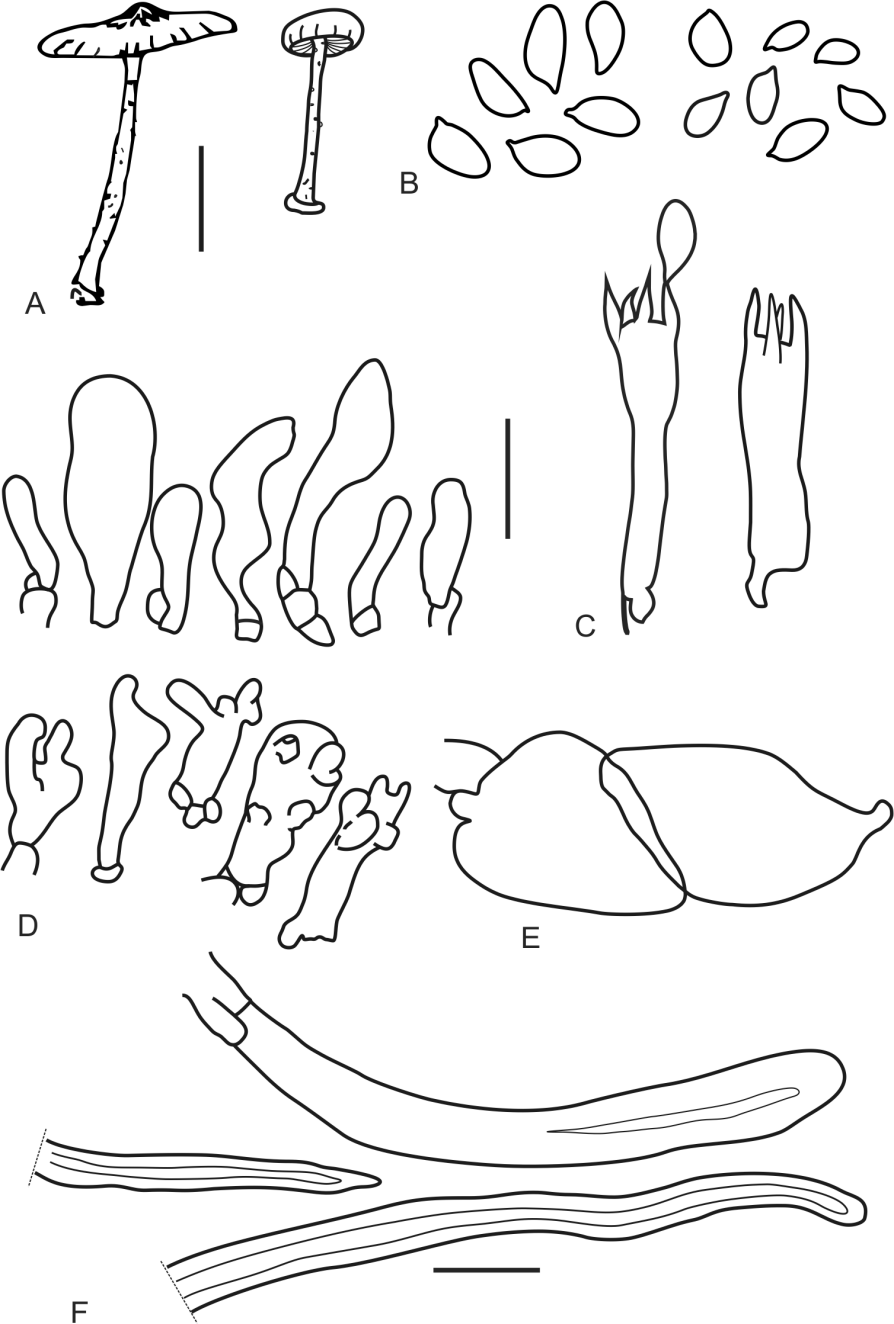
*Moniliophthoramayarum*: **A** basidiomes **B** basidiospores **C** basidium **D** cheilocystidia **E** hypodermium cells **F** pileipellis elements. Scale bars: 10 mm (**A**); 10 µm (**B–F**).

The two Neotropical species are more robust, reaching 20 mm in diameter (or more in *M.ticoi*), compared to the two Paleotropical species, 6–11 mm in *C.hygrocyboides* and 3–15 mm in *M.aurantiaca*. Antonín (2007) published a type revision of *C.hygrocyboides* based on study of an isotype that included microscopic measurements and observations of spores and cheilocystidia as neither Hennings (1902) nor Singer (1989) included these details and Halling (1993) reported he could not find spores or cystidia in the type. The spores of *M.mayarum* are distinctly narrower (3.2–4.2 µm) than those of *C.hygrocyboides* [4.5–6(–7) µm]. While Antonín’s description of the cheilocystidia in *C.hygrocyboides* notes they are ornamented with apically branched obtuse projections, cheilocystidia shape seems to be highly variable and therefore unreliable for distinguishing species in this group. The basidiospores are longer and broader in both *M.aurantiaca* 7.5–11 × 4–6, and *M.ticoi* (9.5–)10.5–13.7 × (3.8–) 4.5–6.3 µm, than in *M.mayarum* 6.5–8.5(–10.5) × 3.2–4.2 µm. Only the larger spores of *M.mayarum* are used in the preceding comparison, and it is not clear why there is a cohort of smaller basidiospores also present. Although, different spore sizes are often observed in the presence of bisporic basidia, lacking clamp-connection and bearing larger spores that are mixed with tetrasporic basidia bearing smaller spores, in repeated examination of the material specifically looking for 2-sterigmate basidia and absence of basal clamps, we observed only 4-sterigmate basidia, and all hymenial elements with clamp-connections. Furthermore, one of the illustrated 4-spored basidia (Fig. 3C), shows a large spore attached to a 4-sterigmate basidium with a basal clamp connection, which negates the hypothesis of a bisterigmate origin for the large spore cohort in *M.mayarum*. Although small spores observed on the hymenium could have been immature and thus smaller, basidiospores of similar size and shape were observed on the pileipellis surface that must have been released from basidia, which indicates they were mature. A similar case occurs in *Crinipellistrinitatis* Dennis. Dennis (1951) in the original description reported smaller basidiospores (5–7 × 2–4 µm) than the revised description of the type by Pegler (1983) (7–9 × 4.1–5.1 µm), so there may be something unusual in the phenology of spore production in this group that leads to two size classes of spores depending on when they are formed and released.

### New combinations

#### Moniliophthora brasiliensis

Taxon classificationFungiAgaricalesMarasmiaceae

(Arruda, G.F.Sepúlveda, R.N.G.Miller, M.A.Ferreira & M.S.Felipe) Niveiro, Lodge & Aime comb. nov.

5F144EBE-43CD-5253-9845-F67D686D4CB3

830320

Genbank No: AY317137

≡ Crinipellisbrasiliensis Arruda, G.F.Sepúlveda, R.N.G.Miller, M.A.Ferreira & M.S.Felipe, Mycologia 97: 1355 (2006). Type: Brazil. Minas Gerais, Itumirim. On dry fan brooms of Heteropterysacutifolia Adr. Juss., 19 Oct 1999, MCC de Arruda 43 [Holotype: UB (Mycol. Col.) 19198].

##### Distribution.

This species is known from Minas Gerais, Brazil (Arruda et al. 2005).

##### Observations.

*Moniliophthorabrasiliensis* is characterized by the light pink to crimson red pileus surface, ellipsoidal basidiospores, 10–14 × 5–7 µm, and lageniform cheilocystidia, with a thin apex, 28–37 × 10–16 µm in size (Arruda et al. 2005). *Moniliophthorabrasiliensis* is a parasite of *Heteropterysacutifolia* (Malpighiaceae). Only ITS sequence data are available for this taxon, which was derived from a dikaryotic basidiome collected from a necrotic broom on *H.acutifolia* (Arruda et al. 2005). *Moniliophthorabrasiliensis* is extremely similar to *M.perniciosa* and diagnosis between the two species at present is based soley on differences in ITS sequence data (Arruda et al. 2005).

#### Moniliophthora ticoi

Taxon classificationFungiAgaricalesMarasmiaceae

(Halling) Niveiro, Ramírez, Lodge & Aime comb. nov.

6CA5B267-9C23-504E-8146-A93CF8C6D779

830321

Genbank No: ITS: MT162721, MT162720. LSU: MT162717, MT162716.

Figs 2B–F , 4A–D

≡ Crinipellisticoi Halling, Mycotaxon 47: 379 (1993). Type: Bolivia. Beni, Iturralde, S of Rurrenabaque, Rio Tuichi near junction with Rio Beni, “Laguna del Tigre”, 14°25'S, 67°30'W, 14 Apr 1990, R.Halling 6433 (Isotype: NY!).

##### Description.

***Pileus*** 7–40(–62) mm, parabolic to convex when young, convex to plane with age, with a shallow umbilicus, surface bright orange (7A8–8A8) with reddish to dark brown center (7C7–7C8), with a narrow light yellowish margin (6A7–6B7 to near 5A6–5A7), dry or moist but not hygrophanous, tomentose or subtomentose in disc, pubescent margin in young specimens, striate disc in young specimens, more marked at the margin, in mature or driest basidiomes with reddish to dark brown sulcate margin (7C8–8C8). ***Lamellae*** subdistant, 1 per mm, adnexed to narrowly adnate, thick and broad, not intervenose, concolorous with the pileus surface (7A8–6A8); edge entire, concolorous with sides, with 2 tiers lamellulae inserted. ***Stipe*** 18–68 × 1–3.5 mm, central, cylindrical, equal or slightly thinner towards the middle, sometimes with a small basal bulb, solid, surface orange to reddish (7A7–7A8) in young specimens, light orange, yellowish orange to creamy yellow (5A6–5A7 to 4A8) and brown (6D8–6D7) toward base in older specimens, densely pubescent at apex when young, then fibrillose-pruinose, dry, insititious. ***Annulus*** absent, but forming a strongly pubescent zone where the veil is inserted in young specimens. ***Spore-print*** not observed, presumably white. ***Context*** pale orange (5A5) in pileus, thin, fleshy in the center and membranous towards the margins, orange white (5A2) in stipe. Odor and taste not tested. KOH and NaOH reactions on pileus surface negative.

**Figure 4.**
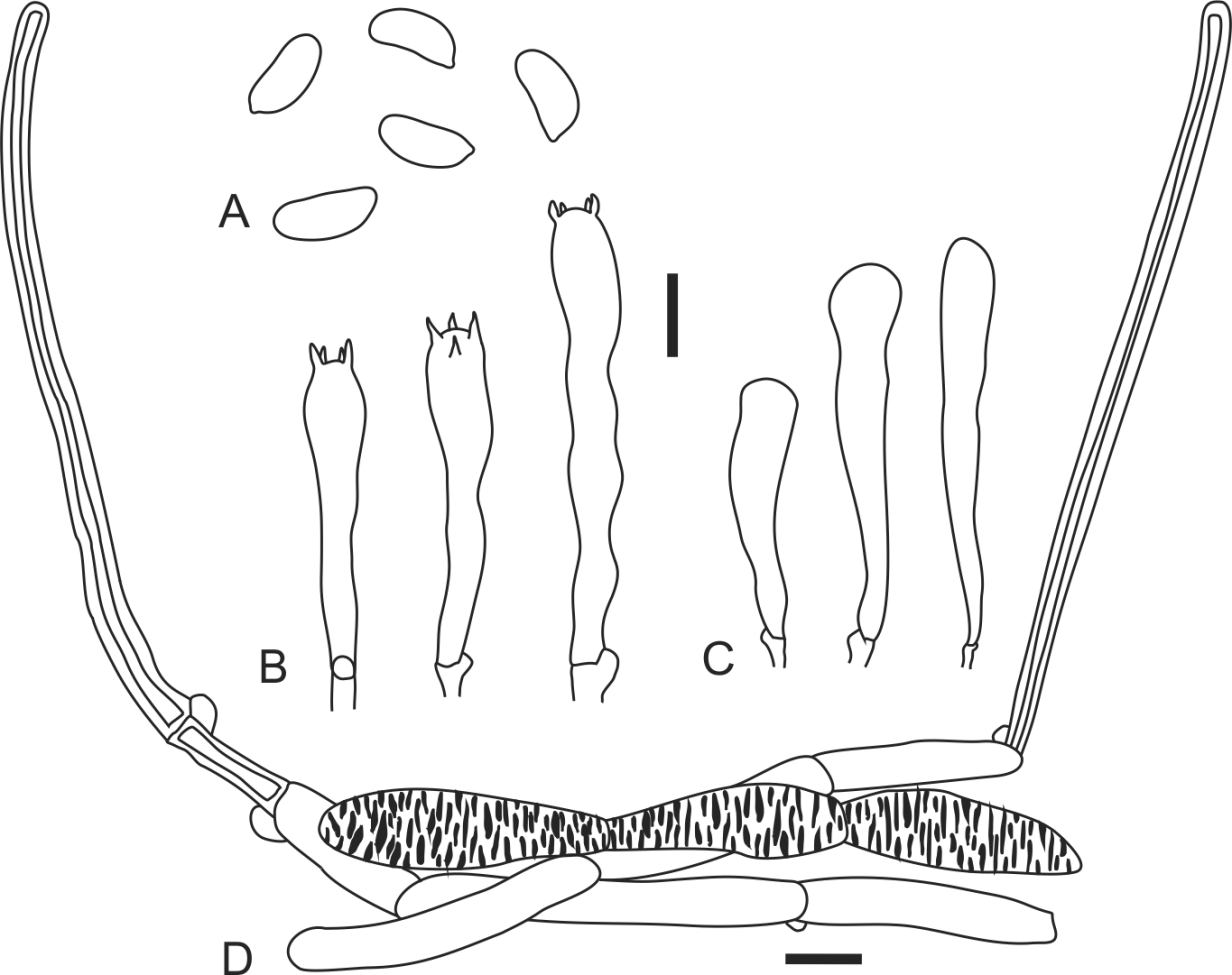
*Moniliophthoraticoi*: **A** spores **B** basidia **C** cheilocystidia **D** pileipellis elements. Scale bars: 10 µm.

***Basidiospores*** (9.5–)10.5–13.7 × (3.8–)4.5–6.3 µm, *x*= 12.1 +/-0.8 × 5.4 +/-0.4 µm; Q= 2.11–2.67; Q*_x_*= 2.38 +/-0.1; n= 60; N=2; oblong to subcylindrical, phaseoliform in side view, thin-walled, smooth, hyaline, inamyloid, without germ-pore. ***Basidia*** 34.3–58 × 7.7–8.6 µm, subcylindrical to narrowly clavate, 4-spored. ***Pleurocystidia*** absent. ***Cheilocystidia*** 32–43 × 7–10 µm, subcylindrical to narrowly clavate, inconspicuous, thin-walled, smooth, hyaline. ***Hymenophoral trama*** subregular, hyphae 40–150 × 5–12 µm, smooth, thin-walled, with clamp-connections. ***Pileipellis*** a cutis of repent, more or less interwoven hyphae, 4–15 µm broad, occasionally with incrusted pigments, covered by clusters of dextrinoid hairs and chains of thin-walled monilioid, inamyloid hyphae. ***Hairs of the pileus surface*** setiform, scattered on the surface, distributed mainly towards the margin, arising from a pileipellis, 90–560 × 4.5–9 µm, dextrinoid, thick-walled, hyphal walls 1.5–3 µm diam, with basal clamp-connection, occasionally 1 or 2 septate, with obtuse apex. ***Stipitipellis*** a cutis of repent hyphae, 6–10 µm broad, with abundant dextrinoid hairs, 40–370 × 5–10 µm, setiform, thick-walled, with obtuse apex, basal clamp-connections.

##### Distribution.

This species is known from Bolivia (Halling 1993) and northern Argentina (Yungas and Chaco region).

##### Ecology.

Gregarious. Parasitic on living roots and trunks of *Myrcianthespungens* (O.Berg) D.Legrand, (Myrtaceae) *Holocalixbalansae* Micheli (Fabaceae) and *Pogonopustubulosus* (A.Rich.) K-Schum (Rubiaceae), in tropical and subtropical forest.

##### Specimens studied.

Argentina • Chaco, 1° de Mayo, Colonia Benitez Educational Reserve, interpretative trail; 27°19'04.12"S, 058°56'59.58"W, 64 m a.s.l.; on Guabiyú (*Myrcianthespungens* – Myrtaceae) trunk and roots; 21.III.2014; N.Ramírez & N.Niveiro CB 23-65 (CTES). • Ibid., on trunk and roots of Alecrín (*Holocalixbalansae* – Fabaceae); 22.III.2016; N.Ramírez & N.Niveiro 103, 105 (CTES). • Jujuy, Ledesma, Calilegua National Park, Guarani trail; 23°45'66.1"S, 064°51'15.0"W, 627 m a.s.l.; on montane forest, on *Pogonopustubulosus* (Rubiaceae); 24.III.2011; N.Niveiro, E.Albertó, B.Lechner & T.Baroni 2249 (CTES). Bolivia • Beni, Iturralde, S of Rurrenabaque, Rio Tuichi near junction with Rio Beni, “Laguna del Tigre”; 14°25'S, 067°30'W; 14.IV.1990; R.Halling 6433 (**Isotype**: NY00511157!).

##### Observations.

This species was described by Halling (1993) from Bolivian specimens. It is characterized by its relatively large, bright orange basidiomes, covered with scattered dextrinoid setiform hairs. The most similar species is *M.mayarum*, which shares morphological characters such as the large basidiomes with bright orange coloration. These two species, however, differ clearly by the smaller spores and by the presence of ornamented cheilocystidia in *M.mayarum*. Another similar species is *M.aurantiaca* from American Samoa (Kropp and Albee-Scott 2012). Both share the orange colored pileus surface with a narrow light yellowish margin. However, they differ in that *M.aurantiaca* has the smaller pileus (3–15 mm broad), smaller basidiospores (7.5–11 × 5–8 µm) and numerous cheilocystidia with several irregular apical appendages resembling fingers (Kroop and Albee-Scott 2012). Another similar species is *C.hygrocybioides* (Henn.) Singer from Africa (Singer 1989), however this is a smaller fungus (pileus 6–11 mm broad) with an umbilicate to papilate pileus that is pilose at the margin (Halling 1993). Based on its morphological characters such as the bright orange pileus surface, *C.hygrociboides* could be included in the genus *Moniliphthora*, but new collections are needed to elucidate its habitat and to obtain sequences and corroborate this hypothesis (currently there are not sequences available for *C.hygrocybioides*).

Other known parasitic Neotropical species are *M.perniciosa*, *C.trinitatis* Dennis and *C.siparunae* Singer. *Moniliophthoraperniciosa*, a destructive parasite of *Theobromacacao*, differs in having smaller basidiomes (pileus up to 25 mm diam) with a red pileus surface and white stipe (Singer 1976; Aime and Phillips-Mora 2005). *Crinipellistrinitatis* has a smaller, red pileus and smaller spores [5–7 × 2–4 µm ss. Dennis (1951) and 7–9 × 4–5 µm ss. Pegler (1983)].

*Crinipellissiparunae* is a widely distributed species that is microscopically similar to *M.ticoi*, especially regarding the range of spore size. However, *C.siparunae* is distinguished by its lilac to brownish lilac pileus surface and by its appendiculate cheilocystidia (Singer 1942, 1976). A taxon thought to be closely related to *C.siparunae*, C.eggersiiPat.var.lilaciceps Singer and described from Amazonian Ecuador shares unornamented cheilocystidia with *C.ticoi*, but it differs from the latter in having a violet to lilac vs orange pileus, and broader basidiospores 6–6.5 × (3.8–)4.5–6.3 µm (Singer 1976; Kerekes and Desjardin 2009). Crinipelliseggersiivar.eggersii, which includes the facultative synonym *Marasmiusvinosus* Speg. described from Argentina, has similar spore dimensions (mostly 11–13 × 5.5–6.3 μm) but differs in having a purple to violet purple pileus and a variety of cheilocystidia shapes (ampullaceous, fusiform, cylindrical or clavate and mostly forked, obtuse or mucronate, sometimes with a subcapitate or capitate apex).

Of the three recent collections in northern Argentina, the specimens of the Yungas forest (Niveiro et al. 2249) closely resemble the original description of *M.ticoi*, with specimens not exceeding 40 mm broad and having a bright red pileus surface (Halling 1993). However, the specimens of the Chaco region differ in having larger basidiomes up to 60 mm broad, and a paler coloration (orange with a yellowish margin), differences that may be due to the drier weather conditions in the Chaco region. Another important difference observed in the Argentinean specimens is the habitat. Halling (1993) found this species growing on rotten wood, however, the new specimens examined were growing on tree trunks and roots of living trees, confirming a biotrophic habit for this species during at least part of its life history.

### Key to striking bright orange *Moniliophthora* and *Crinipellis* species

**Table:** 

1	Biotrophic habit, on diverse hosts. Pileus more than 20 mm diam. Neotropical distribution	**2**
–	Saprotrophic habit. Pileus less than 20 mm diam. Paleotropical distribution	**3**
2	Spores 8.0 +/-1.3 × 3.8 +/-0.3 µm, cheilocystidia clavate or hyphoid, or with 2–3 lobes, or clavate with apical digitate appendages or irregular lumps overall	***M.mayarum***
–	Spores larger, 12.1 +/-0.8 × 5.4 +/-0.4 µm, cheilocystidia simple, inconspicuous, subcylindrical to narrowly clavate, thin-walled, smooth, hyaline	***M.ticoi***
3	Stipitipellis covered by short and moderately thick-walled hairs, resembling setae, 52–85 × 5–10 μm	***M.aurantiaca***
–	Stipitipellis covered with larger (48–180 × 12–18 μm), cylindrical to clavate, thick-walled (up to 3.0 μm), slightly dextrinoid hairs	***C.hygrocybioides***

## Discussion

The addition of these three parasitic species into *Moniliophthora* support a hypothesis of a primarily biotrophic/parasitic habit in this linage of Marasmiaceae. However, nutritional strategies for several species not studied in the present work remain to be definitively ascertained: *M.aurantiaca* was found on woody debris (Kropp and Albee-Scott 2012); *M.conchata* on dead twigs of the liana *Trachelospermumasiaticum* (Siebold et Zucc.) Nakai. (Takahashi 2002) and on fallen twigs of an unidentified liana (Antonín et al. 2014); *M.canescens* on a dead fallen twig of a broad-leaved tree (the Type specimen) and on undetermined dicotyledonous plants (Kerekes and Desjardin 2009); *M.marginata* on undetermined decaying woody stem (Kerekes and Desjardin 2009); and in *M.nigrilineata* the substrate was not specified (Kerekes and Desjardin 2009).

Purple, violet, and red pigments in the pileus combined with a negative (not greenish) reaction with KOH distinguish CrinipellissectionIopodinae (Singer 1976), and these characters are shared with the known basidiome-producing species of *Moniliophthora*. Although no recent collections or sequences are available for other species of CrinipellissectionIopodinae, Kerekes and Desjardin (2009) did show that a specimen identified as C.aff.iopus Singer (the type species of Crinipellissect.Iopodinae) belonged in *Moniliophthora*, although due to a lack of data they were unable to confirm this placement. The current study adds striking orange pigmentation to the suite of characteristics for *Moniliophthora*, as well as confirming a biotrophic habit for the majority of species.

## Supplementary Material

XML Treatment for Moniliophthora mayarum

XML Treatment for Moniliophthora brasiliensis

XML Treatment for Moniliophthora ticoi
